# How Does Long-Term Orientation Influence the Investments of Venture Capitals? Evidence From the Organizational Level

**DOI:** 10.3389/fpsyg.2022.785643

**Published:** 2022-02-16

**Authors:** Tianyi Zheng

**Affiliations:** School of Management, Fudan University, Shanghai, China

**Keywords:** long-term orientation, venture investment, content analysis, industrial popularity, reinvestment, venture stage

## Abstract

Amid great uncertainty along with the possibility of huge returns, venture investment decisions are both technical and artistic. Past studies have paid much attention to the influences of objective factors on venture investment. However, subjective factors have been relatively ignored. As a salient psychological mechanism, temporal focus is of great importance for venture capitalists when making their investment decisions. This study performed content analysis to investigate how temporal focus at the organizational level affects investment decisions of venture capital (VC) firms. The results revealed that VCs with higher level of long-term orientation prefer to invest in less popular industries and ventures in the expansion period. Meanwhile, they are less likely to invest in very new start-ups. Moreover, long-term oriented VCs tend to re-invest in start-ups in their portfolios instead of just shooting once on numerous single start-ups. However, the author did not find any support on preferences of VCs for ventures with high level of human capital.

## Introduction

Venture investment has long been a hot topic in both the entrepreneurship and finance fields (Sahlman and Stevenson, 1985; Hallen and Eisenhardt, 2012; Chircop et al., 2020). With the recognition of unknowable and unpredictable extreme risks (Knight, 1921; De Bondt and Thaler, 1995) as well as possibility of gaining huge returns (Huang, 2018), past studies have differentiated venture investment from classic security investment from several angles. A stream of research has investigated processes and criteria used by venture capitalists (MacMillan et al., 1985; Carpentier and Suret, 2015; Monika and Sharma, 2015), highlighting different evaluation processes and focuses on new ventures. Other studies have separately determined factors influencing whether new ventures can receive investments from venture investors, such as geographic and status distance (Hallen, 2008; Wu, 2016), networks and networking abilities (Hallen, 2008; Hallen and Eisenhardt, 2012), signals or certifications from other organizations (Plummer et al., 2016; Bermiss et al., 2017), and framing and storytelling skills (Martens et al., 2007). Overall, existing knowledge of venture investment proves it to be a non-standardizable, difficult to quantify, challenging, and complicated task (Huang, 2018).

Related studies on entrepreneurs have pioneered the application of the psychological perspective in entrepreneurship academy (Brockhaus, 1980; Sexton and Bowman, 1985; Begley and Boyd, 1987). In early years, scholars investigated the impacts of the Big Five personalities and Hofstede cultural values on the decision of people to become entrepreneurs (Ardichvili and Gasparishvili, 2003; Zhao and Seibert, 2006). Later, some malleable factors, such as self-control, risk-taking, self-efficacy, overconfidence, and narcissism, were observed to have more direct influences on entrepreneurial entry and opportunity realization (Navis and Ozbek, 2016; Obschonka and Stuetzer, 2017). Effectuation, regulatory focus, optimism, and achievement motivation were suggested to be crucial to actions entrepreneurs and strategies of their start-ups (Brockner et al., 2004; Collins et al., 2004; Hmieleski and Baron, 2009; Reymen et al., 2017).

As a consequence, inspired by the opening blackbox of the mindset of entrepreneurs and the relationship between the uncertain decision context and the heuristic cognition process (Mousavi and Gigerenzer, 2014), recent studies on venture investment have turned the focus from objective factors to subjective features, exploring the influence of psychological characteristics and cognitive processes. For example, one of the most salient mechanisms is similarity bias, investors have the tendency to invest in entrepreneurs who have the same features with them, such as same education background, working experience (Franke et al., 2006), and cognitive mode (Murnieks et al., 2011). Moreover, motivational clues, such as passion, commitment (Cardon et al., 2017; Warnick et al., 2018), psychological capital (Anglin et al., 2018), developed identity (Wry et al., 2014), and narratives (Pan et al., 2020) of entrepreneurs are also among the psychologically related factors that affect the cognitive process of investors. Meanwhile, progress has also been achieved concerning subjective factors of venture capitalists, which impact the decision process. Scholars have found evidence that intuition and heuristics are widely used by capitalists and show effectiveness in this extreme uncertain context (Dane and Pratt, 2007; Huang and Pearce, 2015). The feeling of trust (Graebner, 2009) and perception of control (Drover et al., 2014), as well as dispositional affects (Chan and Park, 2013) of investors have also been studied. However, large gaps remain to be filled to unravel the mystery of psychological and cognitive mechanisms of capitalists.

Most studies on venture investment have only applied either the psychological perspective or the organizational perspective, whereas few of them have discussed both. However, as the investment decision process of venture capitals (VCs) usually starts from the individual investigation investors on related materials and ends with the collective consensus of an investment committee integrating all the intelligence of capitalists (Drover et al., 2017), investment decisions at the individual level are not completely consistent with those at the organizational level (Carpentier and Suret, 2015). Thus, psychological mechanisms at the organizational level must be explored.

Combining the psychological perspective and the organizational perspective, this study discusses the influence of the long-term orientation (LTO) of VC organizations on their exhibited investment characteristics. Deriving from temporal theories and the essence of venture investment, the author developed hypotheses positing that higher level of LTO of VC organizations entails larger motivation for them to invest in high-level-human capital, less popular industry, expansion stage ventures and ventures they have invested before. Benefitting from a recent quantitative method of content analysis (Martin, 2016), the author used the articles of VCs’ WeChat official accounts as the source for generating LTO at the organizational level. This study contributes to the sporadic literature adapting the content analysis method and second-hand data source to psychological framework. The results support majority of the hypotheses of the author.

## Hypothesis: Long-Term Orientation and Venture Investment

### Psychological Factors in Venture Investment

Scholars have observed psychological factors as significant influencers of venture investment decision-making at early times. Duxbury et al. (1996) described a personality profile of angel investors with an internal locus of control, high needs for achievement, dominance, and autonomy. In addition, Mason and Stark (2004) mentioned psychological differences of angel investors compared with other types of investors, angel investors have stronger motivation of involvement to acquire “satisfaction and enjoyment from playing a role in the entrepreneurial process,” to be altruistic at times, and to give greater emphasis to subjective factors and gut instinct. Later studies on angel investors focused on their decision-making process, pointing out the use of heuristics, gut feel, and motivational clues as important machanisms (Maxwell et al., 2011; Harrison et al., 2015; Huang and Pearce, 2015; Cardon et al., 2017). In crowdfunding, herd effect and regulatory focuses were found to be influential (Ciuchta et al., 2016; Shen et al., 2020). Meanwhile, few studies have discussed the decision-making of an accelerator as a new organizational format in the entrepreneurial ecosystem. Yang et al. (2020) found that gender role congruity also exists in investment decisions in social impact accelerators. Meanwhile, congruity has been studied not only in terms of gender-role dyads but also in terms of cognitive modes (causation/effecuation) in entrepreneurial investment (Murnieks et al., 2011; Balachandra et al., 2017). Furthermore, studies have suggested that heuristics, gut feel, control motivation, and dispositional affect also matter in entrepreneurial investment decisions (Chan and Park, 2013, 2015; Drover et al., 2014; Huang, 2018). However, few studies have dealt with how a single psychological factor influences the preferences of investors for ventures with different features, especially at the organizational level.

### Literature Review of Long-Term Orientation

Temporal orientation has been found to be an influential psychological mechanism, as “time is essentially in the eye of the beholder (Hall, 1984) and varies across people” (Lin et al., 2019, 3115). Several temporal frameworks elaborate on how people perceive and regard time (Trompenaars and Hampden-Turner, 1998; Souitaris and Maestro, 2010), among which the lens of long-term and short-term orientation has attracted wide interest (Laverty, 1996). It offers an anchor for people to consider and balance the focus on the past, present, and future (Karniol and Ross, 1996; Bearden et al., 2006) to form a dominant temporal logic for decisions and actions (Lumpkin and Brigham, 2011).

Based on the achievements of past studies concerning LTO, it is not only a macro culture dimension at the national level (Hofstede, 2001) but also a multidimensional construct of personal psychology (Lumpkin and Brigham, 2011). In the latest literature, LTO is defined as the “tendency to prioritize the long-range implications and impact of decisions and actions that come to fruition after an extended time period” (Lumpkin et al., 2010). Concern for the future is intuitively and actually a key attribute of LTO (Brigham et al., 2013). However, a more accurate and complete understanding of LTO contains a holistic view of time with extended time horizon valuing both the past and the future instead of only caring for the effects of action in the here and now or the short term (Bearden et al., 2006; Lumpkin and Brigham, 2011). Futurity, continuity, and perseverance are the three components of LTO (Bearden, 2006; Lumpkin and Brigham, 2011).

Decision process is hardly possible to be entirely impartial. The orientations and perceptions of decision-makers are mirrored in their decisions while sifting through and reconciling incomplete ambiguous chaotic information (Hambrick et al., 1996). Thus, in intertemporal choices, LTO seems to be a significant force (Lumpkin and Brigham, 2011) widely discussed in strategic decision-making and family businesses literature. Long-term oriented top executives are willing to pursue interests in a farsighted and inclusive way (Miller and Le Breton-Miller, 2006), operate strategic control rather than financial control (Zahra et al., 2004), accelerate the development and deployment of new products (Yadav et al., 2007; Nadkarni and Chen, 2014), and maintain relationships with stakeholders (Flammer and Bansal, 2017). However, they take fewer strategic risks (Gentry et al., 2016). Meanwhile, LTO also improves the comprehensiveness, speed, and creativity of the strategic decision-making process (Lin et al., 2019) as well as innovative and entrepreneurial actions (Hofstede, 1991; Flammer and Bansal, 2017).

### Long-Term Orientation in Venture Investment

Venture capitals invest in new ventures in order to exit through acquisitions or IPOs with considerable returns. However, outcomes vary in terms of whether they can achieve this goal, how long it takes, and how much they will be paid back. Majority of venture investments fail to generate positive returns, behind which stands the fact that new ventures are always accompanied by unpredictable uncertainties of varying types and temporal distributions (Kollmann and Kuckertz, 2010; Huang, 2018). VCs consider these attributes of ventures and balance different distributions of uncertainties, costs, and gains over time (Souder and Bromiley, 2012) to form their investment strategies. With limited amount of money to allocate in the duration of the funds, they reach an investment decision according to criteria under the implicit guidance of a temporal orientation ranging from short to long (Harrison et al., 2015). Thus, the author proposes that VCs with high level of LTO will demonstrate several features.

#### Human Capital

Prior literature has suggested that qualities of both entrepreneurs and economic attributes are essential in venture investment (Franke et al., 2008). We can distinguish investors according to their priorities of attention on either financial evaluation or qualities of entrepreneurial teams (Knockaert et al., 2010). Human capital acts as an intangible indirect long-term asset of firms (Bena et al., 2017) for ensuring sustainable competitive advantages (Hatch and Dyer, 2004). This notion is especially true in start-ups, as several investors have publicly expressed that entrepreneurs come first in their investment criteria. Ventures with high-level human capital founding teams may not exhibit their advantages at present but instead release strength continuously in the future, especially in the later period (Tzabbar and Margolis, 2017). VCs with high level of LTO are willing to provide more time for entrepreneurs with high potential to turn their human capital advantages into innovative products and economic returns (Symeonidou and Nicolaou, 2018). During this time, they also have the motivation to form long-term relationships and trust with entrepreneurial teams (Flammer and Bansal, 2017; Liu et al., 2019) and provide necessary and useful help. Thus, the author suggests that:

H1: A VC with higher level of LTO keeps a larger percentage of new ventures with high-level human capital founding team in its investment portfolios.

#### Industrial Popularity

Investment trends fluctuate from industry to industry (Zhang et al., 2017). After a distinct improvement emerges in an industry, herd behaviors quickly take over it, with hundreds of imitators watching, learning, and following (Banerjee, 1992), and VCs scramble to pursue any opportunity (Valliere and Peterson, 2004). This pattern raises the uncertainties of the future to a very high level. Start-ups and VCs compete fiercely but have difficulty generating any benefit when the bubble bursts and the market cools down (Zhang et al., 2017, 1370). Therefore, chasing the investment trend does not match the benefits of long-term oriented VCs. The valuation of ventures simultaneously rises high along with rising industrial popularities (Valliere and Peterson, 2004), thus increasing the costs and exit thresholds of VCs in the long run. Conversely, investing in less popular industries can help a VC to establish proactive advantages as well as extend the benefit period (Lumpkin et al., 2010) with quite reasonable costs. This move will also contribute to the formation of reputation as a long-term asset (Wang and Bansal, 2012; Ortiz-de-Mandojana and Bansal, 2016) by differentiating VC from others and pioneering new trends. Moreover, the perseverance attribution of LTO facilitates VCs to concentrate on clear directions and expected future, be patient, and impose self-control to eliminate outside disturbances (Le Breton-Miller and Miller, 2011; Brigham et al., 2013). Thus, the author hypothesizes that:

H2: A VC with higher level of LTO tends to invest in less popular industries.

#### Reinvestment

The author suggests that long-term oriented VCs will have higher tendencies to reinvest in same ventures. First, continuity is one of the dimensions of the LTO construct (Brigham et al., 2013). VCs with high level of LTO expect the future return to be continuous (Lin et al., 2019). Hence, they are less likely to pull out from profitable projects and tend to increase resource commitment (Keil et al., 2009; Souder and Bromiley, 2012). Second, interests in developing a long-term relationship also helps increase the affective commitment of investors to the ventures (Cohen, 2007; Brigham et al., 2013). Contrary to short-term oriented people who are more likely to be opportunists (Bakker and Knoben, 2015), long-term oriented investors tend to have more communication with their invested ventures, especially non-task communication (Lin et al., 2019), to form affective ties. This extra effort can help create a climate of trust between investors and start-ups (Adamson et al., 2003). The willingness and possibilities of reinvestment will then be reinforced by the mutual trust and commitment between high-level LTO investors and their portfolio ventures. Third, expecting the decisions to maintain long-lasting effects on the future, the long-term perspective drives decision-makers to search information broadly beyond the vicinity of the current horizon (Lin et al., 2019) before reaching the first investment decision, thus reducing the possibilities of regret and withdrawal. Lastly, for the pursuit of continuity, long-term oriented decision-makers are thought to be more risk averse (Lumpkin et al., 2010). They may keep requirements on certain levels of liquidity and slack (Gentry et al., 2016), which will help maintain their abilities to deal with uncertainty in the future and to save their portfolio ventures from dilemmas. A famous VC capitalist in China who is a great advocate of long-termism publicly said that “investors need to be more conservative toward risk … we do not have to support ventures by offering too much money at one time. Instead, we ought to offer ‘smart’ money to help start-ups at key points.” One of his most successful investment cases is Meituan. He first invested Meituan in round A and reinvested it in rounds B, C, and E, helping the venture achieve multiple milestones. Thus, phasing the investment instead of completing it all at once may be an effective approach for high LTO VCs to control risks and ensure continuous utilization of the capital. Taking together, the author posits that:

H3: A VC with higher level of LTO keeps a larger percentage of new ventures in which it invested before in its investment portfolios.

#### New Venture Stage

As mentioned above, long-term oriented organizations and decision-makers tend to be proactive actors (Lumpkin et al., 2010). The literature on strategic decisions has also suggested that LTO is positively correlated with the speed of introduction of new products (Nadkarni and Chen, 2014) and long-term relationship with stakeholders (Lin et al., 2019). This suggestion may indicate that long-term oriented VCs will form investment ties with start-ups as early as possible. However, they may avoid investing too early for one reason: given the pursuit of continuity, long-term oriented organizations exhibit less willingness to take risk (Gentry et al., 2016), seeing that allowing other risks that amplify temporal risk is not a wise decision (Lin et al., 2019). Meanwhile, investing in ventures in a very early stage may expose VCs to high risks of venture failure and liquidity, as early-stage startups have higher level of ambiguities and uncertainties (Hopp and Lukas, 2014, 643) and lower possibilities of quick exits (Lahr and Mina, 2014). Hence, VCs with high level of LTO may need to balance proactiveness and uncertainties by selecting the appropriate stage to invest. Thus, the author supposes that:

H4: A VC with higher level of LTO keeps a larger percentage of new ventures in the expansion stage and a smaller percentage of ones in the early or mature stage in its investment portfolios.

## Materials and Methods

### Sample and Data Source

As mentioned above, the author performed content analysis to measure the LTO of VCs, and first collected a list of VC organizations from IT Juzi (Su and Lichun, 2020), a website widely used by investors in China. Similar to VentureXpert, it aims to provide the most complete data on Chinese entrepreneurship and investments. It offers three streams of data: (1) founding information on new ventures, including founding dates, founding teams, and descriptions of business ventures; (2) VC profiles, including the amount of their managed money, employees, and other fundamental information; (3) funding information, including investor(s) and invested venture as well as investment date and amount of money. Specifically, the database contains founding and investing data for more than 200,000 ventures, 120,000 investment events, and 10,000 investment organizations, greatly exceeding data volume of other databases (e.g., another frequently used database named PE Daily records only approximately 23,000 investment events). The author matched these VCs with their WeChat official accounts if available. Nowadays, as digital technologies have reformed communication channels, organizations have been using these advancements to build their images and compete for the attention of the audience on the internet. WeChat has become the dominant communication tool and an important media in China. It has also become the most important platform for information dissemination. Organizations can create official accounts on WeChat. Their accounts serve as billboards for organizations to communicate with their audience, release latest news, and promote their ideas. For example, one VC posted an article to promote its investment logic:

*“… In terms of early-stage projects, our advantage is not the amount of capital but the fact that we can do a lot with a little. We look at projects with a keen eye. Early-stage projects are indeed risky, but the high growth of a particular project will cover the loss of other projects…Entrepreneurship and investment are subject to various risks and challenges. Challenges are good, and risks are not necessarily bad. I am partial to investing in higher-risk angel rounds, especially those with resources…”*

The author collected articles published on the WeChat official account of VCs by year using frequencies of LTO keywords in these articles to indicate the level of LTO of VCs. The details are introduced in the latter part of this article.

The matched sample included 606 VCs with 1,473 VC-year observations of LTO from 2012 to 2019^1^. The author collected other data concerning general information and investment records of VCs from IT Juzi that were supplemented by other databases (such as PE daily and Tianyancha) and official websites of VCs.

### Content Analysis and Measure of Long-Term Orientation

The majority of past studies used questionnaires to measure LTO, implying the same premise that the time orientation of top executives represents this aspect of the organization (Hambrick and Mason, 1984). However, the studies seem to evade the question of how to weigh the influence of each executive on organizations under varying levels of LTO (Lin et al., 2019). Thus, it is meaningful to search for direct measurement of such psychological constructs at the organizational level.

Prior research has used firm disclosures (such as annual reports and letters for shareholders) to indicate their visions and intentions (Gerstner et al., 2013; Flammer and Bansal, 2017). With the growing acceptance of cognitive-linguistic theory, content analysis has got wider use in entrepreneurship literature (Lee and Huang, 2018; Pan et al., 2020). According to the Whorf–Sapir hypothesis (Whorf, 1940; Sapir, 1944), thoughts of individuals and organizations (attention, cognitive categories, priorities, etc.) are reflected in the words and vocabularies they use. In this vein, the author attempted to use the method of content analysis to measure the LTO of VCs with frequencies of LTO words used.

Flammer and Bansal (2017) constructed a simple vocabulary of LTO with only four pairs of words: “long run,” “long-run,” “long term,” and “long-term.” Nonetheless, the other dimensions of LTO, except for futurity, were ignored. Then, the author followed the method of Pan et al. (2020) to create a dictionary (vocabulary) with a complete indication of LTO constructs and tested its validity.

Given that a Chinese sample was used, the author applied the simplified Chinese dictionary of LIWC as the basic dictionary, because it includes all commonly used words and is widely accepted in academic research (Parhankangas and Renko, 2017)^2^. First, a PhD student and the author, equipped with theoretical bases of LTO, independently selected keywords representing the construction of LTO from the basic dictionary using the scales from Bearden et al. (2006) as reference. These keywords constituted the original dictionary of LTO. In the second step, five PhD holders/PhD students independently voted on each keyword in the original dictionary, gauging whether the word reflected the meaning of LTO. They were also asked to make their judgments in accordance with the same scale. In the last step, the author calculated the content-validity ratio (CVR) for each keyword. The formula is:


$$\text{CVR}=\frac{n-\text{N}/2}{\text{N}/2},$$

where *n* denotes the votes each word obtained from the judges ranging from 0 to 5, and N is the number of judges (5 in this study). The CVR ranges from −1 to 1.0, and the author only retained keywords with a CVR greater than or equal to 0.8 (which means it received at least four out of five votes from the judges). Finally, the author used 44 keywords to construct the final LTO dictionary.

To test the validity of the LTO dictionary, the author used a random subsample containing 149 articles from the full sample collected. The author and the PhD student who joined the first step to create the original dictionary rated these articles on a five-point Likert scale, with 1 indicating “shows no LTO of VC at all” and 5 indicating “heavily shows the LTO of VC.” Meanwhile, the author measured the LTO indexes of the articles using the LIWC dictionary approach, dividing the total number of LTO keywords by the total number of words of each article. The author calculated ICC = 0.83, which supported the good validity of the linguistic measure of LTO.

### Measures

#### Long-Term Orientation

The author finally measured LTO at the VC-year level by calculating the means of the LTO index of each article the VC posted on its WeChat official account in every focal year. The formulas are as follows:


$$LTOindex_{k}=\frac{thenumberofLTOkeywords_{k}}{thetotalnumberofwords_{k}}\times 100,$$

$$LTO_{vc,year}=\frac{\sum_{1}^{n}LTOindex_{k}\left(vc,year\right)}{n},$$

where *LTO index*_*k*_ represents the LTO index of each article, and *n* represents the number of articles the *VC* posted on its WeChat offical account in a focal year.

#### Human Capital

The author coded the experience of entrepreneurial team members to access the human capital of new ventures and recognized four types of experiences as sources of human capital, educational experience, industrial working experience, management experience, and entrepreneurial experience. The author first measured the human capital of each entrepreneurial team member by one count variable indicating educational experience and three dummies indicating the other three types of experience. For educational experience, the variable was 0 if the member had no degree or lower degree than a bachelor’s degree, 1 for bachelor’s degree, 2 for master’s degree, and 3 for doctoral degree. For industrial working experience, the dummy was equal to 1 if the member had worked in the same industry before he found the venture, otherwise it was 0. For management experience, the dummy was equal to 1 if the member had been a senior manager or a top executive of firms (leader of a department/division/branch instead of a team/group) before. For entrepreneurship experience, the dummy was equal to 1 if the member had founded a start-up or had been a founding partner of a start-up. After that, the author integrated these measures to team-level human capital by calculating the sum of the means of the four variables. Then, the author determined a venture with high-level of human capital if its entrepreneurial team human capital was higher than the average of all the ventures’ received investment in the focal year. Lastly, the author derived the percentage of high-level human capital ventures in its investment portfolios by dividing the number of high-level human capital the VC invested in by the total number of ventures it invested in the focal year.

#### Industrial Popularity

The author followed Zhang et al. (2017) to derive the popularity of a focal industry as the total number of ventures receiving their first VC funding before or in D-round (namely the early and expansion stages) in this industry in the previous year. Then, the author calculated the average industrial popularity of the investment portfolios of VCs in the focal year.

#### Reinvestment

The author calculated the percentage of new ventures that a focal VC had invested in before this focal round in its investment portfolio by dividing the number of ventures that the focal VC had invested in before by the total number of ventures it invested in the focal year.

#### New Venture Stage

The author categorized the ventures according to their funding rounds when they received the investment. The “Seed,” “Angel,” “Pre-A/A/A+,” and “Pre-B/B/B+” rounds represented the early stages, the C and D rounds represented the expansion stages, and the later rounds represented the mature stages. The author calculated the percentage of investments of the early/expansion/mature stage in VCs’ investment portfolios separately by dividing the number of corresponding ventures the VC invested in by the total number of new ventures it invested in the focal year.

#### Controls

Following the prior venture investment literature, the author controlled the number of investors (invest analysts), age, amount of money managed with log transformation, and number of branches of each VC, which were indications of its experience and capability and might impact its investment behaviors. The author also controlled the number of foreign branches to control for more diverse information sources of the VC. Moreover, the author controlled the investment experience of the VC by the number of investments made in the 5 years before the focal year. The author controlled the investment accomplishments of the VC by the number of ventures it invested in the 5 years before the focal year by the VC that went to IPO later. The author controlled the number of industries in which the VC invested in the 5 years before the focal year to eliminate the possible influence of the industrial horizon of the VC. Lastly, as the network is supported as a strong force for investment decision (Zhelyazkov, 2018), the author controlled the effect of the network of the VC by the number of VCs that invested together with the focal VC in the 5 years before the focal year. The author used the log transformation of investment experience and network to adjust for skewness and to capture non-linear impacts. In addition, the author controlled the fixed effects of calendar years and cities where headquarters were located as well.

## Results

### Data Description

Tables 1, 2 show the description and correlations of the variables. LTO did not exhibit any high correlations with the dependent variables and control variables, whereas some of the controls were correlated with one another. *Investment experience* was highly correlated with *Industrial horizon* and *Network* of VCs, which was understandable, as the more investments a VC made, the higher possibilities for it to invest in different industries and form ties with other VCs. *Network* also had a high correlation with *Industrial horizon*, as a larger network could offer more information and knowledge for the VC to explore opportunities in diverse industries. High correlations (above 0.5) appeared in other dyads among *Number of investors*, *Investment experience*, *Investment accomplishments*, *Industrial horizon*, and *Network* as well. The remaining controls, namely, *Number of branches*, *Number of foreign branches*, *Age*, and *Amount of money managed*, were not highly correlated with each other. The author applied OLS regression to test the hypotheses as expressed by Equation (1) below:


(1) $$DV_{i,t}=\beta LTO_{i,t}+\delta Controls_{i,t}+p_{i}+y_{t}+\varepsilon_{i,t}$$

where *i* represents each VC in the sample, and *t* represents each calendar year. Thus, *DV_*i,t*_, LTO_*i,t*_*, and *Control*_*i,t*_ represent the dependent variables, LTO value, and control variables of each observation, respectively. *p*_*i*_ and *y*_*t*_ are the fixed effects of places and calendar years. As the sample is of the VC-year level, the author clustered the errors to the VC level in the estimation.

**TABLE 1:** Statistics of variables.

Variable	Obs.	Mean	SD	Min	Max
LTO	1473	0.486	0.218	0	1.972
Percentage of high-level human capital new ventures	1473	0.488	0.347	0	1
Industrial popularity of portfolio	1473	0.035	0.021	0.002	0.126
Percentage of new ventures invested before	1473	0.105	0.197	0	1
Percentage of investments at early stage	1473	0.823	0.292	0	1
Percentage of investments at expansion stage	1473	0.08	0.188	0	1
Percentage of investments at mature stage	1473	0.096	0.228	0	1
Number of investors	1473	8.432	6.567	1	38
Investment experience	1473	2.502	1.189	0.693	5.756
Investment accomplishments	1473	1.197	2.767	0	23
Industrial horizon	1473	6.964	5.329	1	20
Network	1473	2.630	1.288	0	5.858
Number of branches	1473	1.666	1.426	1	11
Number of foreign branches	1473	0.058	0.315	0	4
Age	1473	4.924	4.229	0	31
Amount of money managed	1473	22.103	1.637	16.588	26.022

**TABLE 2:** Correlations.

Variables	LTO	Percentage of high-level human capital new ventures	Industrial popularity of portfolio	Percentage of new ventures invested before	Percentage of investments at early stage	Percentage of investments at expansion stage	Percentage of new ventures at mature stage
Percentage of high-level human capital new ventures	0.053**						
Industrial popularity of portfolio	−0.060**	0.075***					
Percentage of new ventures invested before	0.116***	0.014	−0.026				
Percentage of investments at early stage	−0.082***	0.060**	−0.055**	0.015			
Percentage of investments at expansion stage	0.089***	0.007	0.044*	0.057**	−0.628***		
Percentage of investments at mature stage	0.031	−0.084***	0.032	−0.067**	−0.765***	−0.022	
Number of investors	0.055**	−0.010	0.022	0.111***	−0.029	0.027	0.014
Investment experience	0.100***	0.010	0.008	0.235***	0.044*	0.021	−0.075***
Investment accomplishments	0.061**	−0.028	−0.022	0.104***	−0.067**	0.076***	0.023
Industrial horizon	0.115***	−0.028	−0.068***	0.207***	0.065**	0.003	−0.086***
Network	0.144***	0.030	0.039	0.260***	−0.060**	0.119***	−0.023
Number of branches	0.012	−0.005	−0.001	0.069***	0.027	−0.017	−0.021
Number of foreign branches	0.032	0.004	0.007	0.027	0.024	−0.001	−0.030
Age	0.008	−0.025	0.100***	0.057**	−0.106***	0.055**	0.091***
Amount of money managed	0.077***	0.024	0.088***	0.019	−0.132***	0.088***	0.097***

Variables	Number of investors	Investment experience	Investment accomplishments	Industrial horizon	Network	Number of branches	Number of foreign branches	Age
Investment experience	0.573***							
Investment accomplishments	0.541***	0.549***						
Industrial horizon	0.568***	0.783***	0.523***					
Network	0.521***	0.884***	0.530***	0.727***				
Number of branches	0.251***	0.193***	0.186***	0.256***	0.162***			
Number of foreign branches	0.067***	0.017	0.116***	0.065**	0.067**	0.397***		
Age	0.439***	0.279***	0.331***	0.301***	0.318***	0.154***	0.098***	
Amount of money managed	0.269***	0.139***	0.225***	0.143***	0.169***	0.139***	0.120***	0.277***

***p < 0.01, **p < 0.05, *p < 0.1.

### Main Models

The results of the regression models are displayed in Tables 3, 4. The results did not support hypothesis 1, as the LTO coefficient in the model of H1 was not significant [β = −0.016, *t* = −0.34, *p* = 0.732, and 95% CI = (−0.108, 0.076)]. As for the models of H2, the LTO showed significant negative effects on the industrial popularity of the portfolio [β = −0.008, *t* = −2.72, *p* = 0.007, and 95% CI = (−0.014, 0.002)]. Thus, hypothesis 2 was supported, that is, VCs with higher level of LTO tend to have less “popular” portfolio. Hypothesis 3 was also supported by a significantly positive coefficient of LTO [β = 0.056, *t* = 2.54, *p* = 0.011, and 95% CI = (0.013, 0.099)], which indicated that VCs with higher level of LTO tend to invest in the same venture continuously instead of shooting once at numerous single ventures. The models of hypothesis 4 containing three regressions demonstrated the expected significant effects of LTO on the percentage of investments in the early and expansion stages [early stage: β = −0.068, *t* = −1.92, *p* = 0.056, and 95% CI = (−0.137, 0.002); expansion stage: β = 0.052, *t* = 2.22, *p* = 0.027, and 95% CI = (0.006, 0.098)]. However, LTO showed no significant effect on the percentage of investments in the mature stage [β = 0.015, *t* = 0.51, *p* = 0.608, and 95% CI = (−0.044, 0.075)]. This result suggested that VCs with higher level of LTO are more interested in investing in ventures in the expansion stage but more conservative in ventures in the early stage. In addition, they maintain a neutral attitude toward mature ventures. Thus, hypothesis 4 was partially supported.

**TABLE 3:** Regression results of H1 and H2.

	H1		H2	
Variables	Percentage of high-level human capital new ventures		Industrial popularity of portfolio	
LTO		−0.0161		−0.00789**
		(0.0469)		(0.00290)
Number of investors	0.000959	0.000966	2.81e-05	3.13e-05
	(0.00206)	(0.00206)	(0.000124)	(0.000124)
Investment experience	0.00753	0.00732	0.0111***	0.0110***
	(0.0285)	(0.0286)	(0.00188)	(0.00187)
Investment accomplishments	−0.00504^+^	−0.00500^+^	−0.000162	−0.000141
	(0.00269)	(0.00269)	(0.000185)	(0.000183)
Industrial horizon	−0.00817^+^	−0.00814^+^	−0.00273***	−0.00272***
	(0.00466)	(0.00466)	(0.000394)	(0.000388)
Network	0.0393*	0.0397*	0.000272	0.000461
	(0.0173)	(0.0175)	(0.00106)	(0.00105)
Number of branches	−0.00196	−0.00190	−5.74e-05	−3.27e-05
	(0.00632)	(0.00632)	(0.000483)	(0.000494)
Number of foreign branches	0.00624	0.00642	0.000782	0.000871
	(0.0313)	(0.0314)	(0.00155)	(0.00151)
Age	−0.00253	−0.00260	0.000382*	0.000344*
	(0.00267)	(0.00268)	(0.000157)	(0.000155)
Amount of money managed	0.00571	0.00586	0.000865*	0.000939*
	(0.00608)	(0.00608)	(0.000388)	(0.000386)
Constant	0.467**	0.463**	0.0182*	0.0163^+^
	(0.177)	(0.177)	(0.00867)	(0.00866)
Observations	1,473	1,473	1,473	1,473
R-squared	0.057	0.057	0.181	0.187

***p < 0.001, **p < 0.01, *p < 0.05, ^+^p < 0.1.

**TABLE 4:** Regression results of H3 and H4.

	H3		H4					
Variables	Percentage of new ventures invested before		Percentage of investments at early stage		Percentage of investments at expansion stage		Percentage of investments at mature stage	
LTO		0.0559*		−0.0678^+^		0.0518*		0.0154
		(0.0220)		(0.0354)		(0.0233)		(0.0301)
Number of investors	−0.000102	−0.000125	−0.000743	−0.000715	−0.000681	−0.000702	0.00138	0.00138
	(0.00107)	(0.00106)	(0.00173)	(0.00173)	(0.00109)	(0.00109)	(0.00154)	(0.00154)
Investment experience	0.106***	0.107***	0.102***	0.101***	−0.0249	−0.0243	−0.0772***	−0.0770***
	(0.0172)	(0.0170)	(0.0281)	(0.0280)	(0.0176)	(0.0176)	(0.0227)	(0.0227)
Investment accomplishments	−3.70e-06	−0.000151	−0.0127**	−0.0125**	0.00648**	0.00635**	0.00620*	0.00616*
	(0.00212)	(0.00211)	(0.00424)	(0.00425)	(0.00219)	(0.00218)	(0.00300)	(0.00300)
Industrial horizon	−0.0160***	−0.0161***	0.00657^+^	0.00669^+^	−0.00639*	−0.00648*	−0.000134	−0.000159
	(0.00340)	(0.00334)	(0.00397)	(0.00396)	(0.00263)	(0.00263)	(0.00310)	(0.00310)
Network	0.00746	0.00612	−0.0943***	−0.0927***	0.0517***	0.0504***	0.0425**	0.0421**
	(0.0108)	(0.0108)	(0.0173)	(0.0171)	(0.0112)	(0.0110)	(0.0132)	(0.0132)
Number of branches	0.00757	0.00739	−0.00504	−0.00482	0.000712	0.000550	0.00436	0.00431
	(0.00480)	(0.00462)	(0.00514)	(0.00506)	(0.00311)	(0.00300)	(0.00440)	(0.00439)
Number of foreign branches	0.00739	0.00676	0.0572*	0.0579*	−0.0117	−0.0123	−0.0455*	−0.0456*
	(0.0159)	(0.0162)	(0.0272)	(0.0274)	(0.0155)	(0.0155)	(0.0179)	(0.0180)
Age	−0.00111	−0.000845	−0.00357^+^	−0.00389^+^	−3.71e-05	0.000211	0.00358^+^	0.00365^+^
	(0.00132)	(0.00133)	(0.00215)	(0.00214)	(0.00131)	(0.00128)	(0.00202)	(0.00203)
Amount of money managed	−0.000860	−0.00139	−0.0170**	−0.0163**	0.00731*	0.00683^+^	0.00966^+^	0.00951^+^
	(0.00353)	(0.00348)	(0.00598)	(0.00599)	(0.00352)	(0.00354)	(0.00504)	(0.00505)
Constant	−0.204*	−0.190*	1.177***	1.160***	−0.0941	−0.0814	−0.0835	−0.0797
	(0.0801)	(0.0790)	(0.135)	(0.135)	(0.0846)	(0.0852)	(0.111)	(0.111)
Observations	1,473	1,473	1,473	1,473	1,473	1,473	1,473	1,473
R-squared	0.141	0.144	0.139	0.141	0.077	0.081	0.095	0.095

***p < 0.001, **p < 0.01, *p < 0.05, ^+^p < 0.1.

In addition to the main findings, the author also observed some significant coefficients of the control variables. The industrial horizon showed negative effects on all the dependent variables except for the new ventures in the early stage, possibly because VCs pursuing industrial diversity of their investment portfolios may reduce their attention on other attributions of ventures, such as human capital and popularity. Moreover, in order to invest in a larger industrial range, VCs may need to separate their money into pieces to invest more ventures in different industries. Thus, they may lack concentration on single ventures and attempt to catch opportunities from the early stage with fewer expenses. Conversely, a larger network of VCs results in more investments in high-level human capital new ventures as well as new ventures in the extension and mature stages. This outcome may be due to the information and knowledge spillover from the other members of the network.

### Robustness Check

For robustness check, the author first ran the models with all variables to be standardized. The results turned out to be almost consistent. For H2, H3, and H4, which were supported in the main models, the author carried out extra models for robustness check.

For H2, which was about industrial popularity, the author applied a new measure to capture the industrial popularity level of the portfolios, which was similar to the measure of human capital explained in the Section “Materials and Methods.” The author calculated the mean of the popularities of industries yearly and determined a venture as popular if it belonged to an industry with higher popularity than the mean. Then, the author accessed *the percentage of high-popularity new ventures* by dividing the number of *popular ventures* the VC invested in by the total number of ventures it invested in the focal year.

For reinvestment (H3), the author calculated the time a venture invested before the focal year by the focal VC (if a venture was first invested by the focal VC in the focal year, it was equal to 0). Then, the author integrated them to the portfolio level by calculating the means across portfolio ventures of a VC on each focal year as the measure of the focal VC’s preference for reinvestment.

As for the hypothesis of preference on stages (H4), the author substituted the classified measures of stages with a continuous one. The author first coded the *round* by sequence: “Seeds” is equal to 0, “Angel” is equal to 1, “Pre-A/A/A+” is equals to 2, “Pre-B/B/B+” is equal to 3, “C/C+” is equal to 4, “D/D+” is equal to 5, “E” is equal to 6, “F to before-IPO” is equal to 7, and “after IPO” is equal to 8. Then, the author calculated *the average round of each VCs’ portfolio ventures* yearly, the higher of which indicated the tendency of a VC to invest in more mature ventures.

**FIGURE 1:**
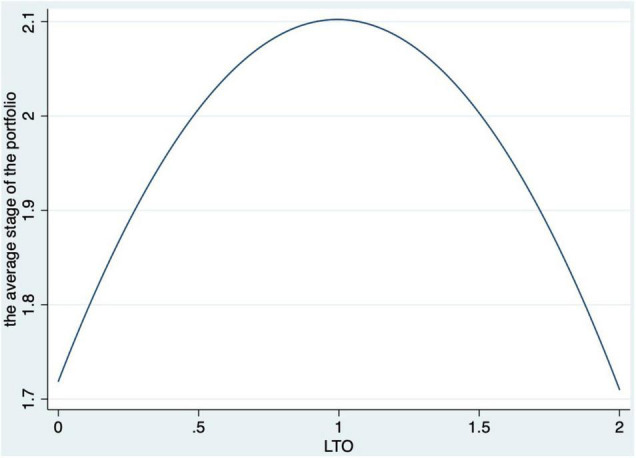
Inverted “U”-shaped relationship between long-term orientation (LTO) and preference of investment stage.

The author reports the additional results of the robustness check in Table 5. For H2 (industrial popularity) and H3 (re-investment), the significant coefficients of LTO again supported the two hypotheses [H2: β = −0.095, *t* = −2, *p* = 0.046, and 95% CI = (−0.189, −0.002); H3: β = 0.088, *t* = 2.95, *p* = 0.003, and 95% CI = (0.030, 0.147)]. One percent rise in the VC’s LTO may cause a 9.5 decline in the percentage of high-popularity ventures in its portfolio and a 0.088 increase in the frequency of investing in the same venture. For H4, which proposed that LTO is first positively related to preference in later stages and becomes negative after, the author tested the possible inverted “U”-shaped relationship between LTO and preference of the investment stage by introducing the squared term of LTO, as shown in Equation (2). Consequently, the inverted “U”-shaped relationship was supported, as the coefficient of LTO was significantly positive while the coefficient of the squared term of LTO was significantly negative [LTO: β = 1.125, *t* = 3.85, *p* = 0, and 95% CI = (0.551, 1.699); LTO^2: θ = −0.554, *t* = −2.42 *p* = 0.016, and 95% CI = (−1.003, −0.105)]. The inverted “U” shape is clearly visible in Figure 1. This outcome confirmed our hypothesis that VCs with higher LTO prefer ventures in the expansion stage over those in the early and mature stages.


(2) DVi,t=θLTOi,t^2+βLTOi,t+δControlsi,t+pi+yt+εi,t.

**TABLE 5:** Robustness check for H2, H3, and H4.

	H2		H3		H4	
Variables	Average popularity of the portfolio ventures		Average invested times by focal VC of the portfolio ventures		Average round of the portfolio ventures	
LTO		−0.0952*		0.0884**		1.125***
		(0.0477)		(0.0300)		(0.292)
LTO^2						−0.554*
						(0.229)
Number of investors	0.000512	0.000551	−0.000122	−0.000158	0.00414	0.00391
	(0.00226)	(0.00226)	(0.00147)	(0.00144)	(0.00723)	(0.00721)
Investment experience	0.191***	0.190***	0.132***	0.133***	−0.502***	−0.494***
	(0.0325)	(0.0324)	(0.0223)	(0.0219)	(0.109)	(0.107)
Investment accomplishments	−0.00225	−0.00200	0.000354	0.000121	0.0487***	0.0473***
	(0.00333)	(0.00330)	(0.00301)	(0.00298)	(0.0142)	(0.0142)
Industrial horizon	−0.0464***	−0.0462***	−0.0195***	−0.0196***	−0.0523***	−0.0538***
	(0.00594)	(0.00586)	(0.00467)	(0.00456)	(0.0145)	(0.0144)
Network	−0.00192	0.000369	0.0138	0.0117	0.525***	0.509***
	(0.0204)	(0.0203)	(0.0121)	(0.0122)	(0.0647)	(0.0638)
Number of branches	−0.00177	−0.00147	0.00870	0.00843	0.0640**	0.0630**
	(0.00781)	(0.00799)	(0.00667)	(0.00641)	(0.0217)	(0.0218)
Number of foreign branches	0.0444	0.0455^+^	−0.000879	−0.00188	−0.193^+^	−0.196^+^
	(0.0276)	(0.0274)	(0.0178)	(0.0182)	(0.106)	(0.110)
Age	0.00737*	0.00692*	−0.00249	−0.00207	0.0220*	0.0246**
	(0.00296)	(0.00294)	(0.00160)	(0.00159)	(0.00952)	(0.00936)
Amount of money managed	0.0170*	0.0179*	−0.00167	−0.00250	0.0647**	0.0598**
	(0.00742)	(0.00735)	(0.00504)	(0.00497)	(0.0216)	(0.0214)
Constant	0.131	0.107	−0.250*	0.185	0.317	0.673^+^
	(0.164)	(0.162)	(0.115)	(0.479)	(0.476)	(0.400)
Observations	1,473	1,473	1,473	1,473	1,473	1,425
R-squared	0.157	0.160	0.161	0.203	0.212	0.272

***p < 0.001, **p < 0.01, *p < 0.05, ^+^p < 0.1.

## Discussion

In this study, the author discussed the essence of LTO in venture investment. Concentration on the future, perseverance, and pursuit for continuity (Lumpkin and Brigham, 2011) influence venture investment at the organizational level. The empirical analysis strongly suggested that VCs with high level of LTO are more likely to put their money in less popular industries for the sake of forming longer-lasting proactive advantages. Instead of putting eggs in as many baskets as possible, VCs with high level of LTO have more patience in and dedication to their invested ventures with higher possibility of re-investing in a single venture. Lastly, the author proved the high LTO preference of VCs for ventures in the expansion stage. It may be intuitively suggested that the concentration on long-term benefits leads VCs to invest as early as possible. However, the impact of LTO on the timing of investment turned out to be a balanced result of the pursuit of long-term benefits and excessive risk aversion.

The author did not find support for any preference of high-level LTO VCs to high-level human capital of founding teams (H1). One possible speculation is that long-term oriented investors may be more interested in the role of a “coach” (Colombo and Grilli, 2010) who can contribute more to the development of ventures. They are willing to put their eyes on inconspicuous ventures or entrepreneurs at present yet with qualities and a high potential for future growth under their coaching. This notion coincides with the logic behind the industry popularity hypothesis in this study. Additionally, the different sources of human capital may be related to different levels of coachabilities, which may be valued by VCs differently. The author suggests that the relationship between characteristics of entrepreneurs and their coachabilities may be an interesting topic for future research.

One of the features of this study is performing content analysis for LTO measure. Content analysis has become a popular method for cognition-related research (Gerstner et al., 2013; Pan et al., 2020). By the analysis of officially released articles, the author generated the LTO measure directly at the VC organizational level, thus testing the psychological framework at the organizational level. This pioneered the psychological and cognitive empirical research to take advantage of second hand data of larger size.

Scholars have long called for cognitive exploration in the field of entrepreneurship (Shepherd and Patzelt, 2018). However, the mindset of investors is still covered by mist. Thus, this study has extended venture investment research by deepening the psychological view in this field. Along with the validation of LTO theory in the venture investment context, this study also serves as a reference for strategic decision-making research, as the context of venture investment decision is sometimes considered an extreme approximate of strategic decision-making (Maitland and Sammartino, 2015). Strategic decisions of firms are usually related to large long-term investments in projects, assets, or M&As where uncertainties and lack of information also stimulate the heuristic process (Loock and Hinnen, 2015). Moreover, VC firms have diverse strategies guiding the formation of their portfolios (Drover et al., 2017). Thus, the mechanism of venture investment may also be valid in strategic decision contexts.

The conclusion of this study provides a practical lens for VCs to examine their investment decisions from the angles of both process and result. Venture investors are suggested to understand their subjective decision process based on the understanding of their mindsets. Meanwhile, VCs need to consider the psychological characteristics of their employees to execute specific investment strategies. For entrepreneurs, this revealed mechanism should also be considered when selecting desired investors.

### Limitations and Future Research

This study is limited in several ways. First, the author did not test the moderate effects of other related factors. Evidence shows that affects and cognitions are always intertwined in the decision making process (Elen et al., 2013). Future research can contribute by testing whether their interplay still exists at the organizational level. In addition, environmental factors can serve as important contingencies (Nadkarni et al., 2016; Lin et al., 2019) despite the challenge that venture investors may react to the environment much differently from top executives, as they have to look across industries and quickly catch the dynamics. Another deficiency is that the author did not have the opportunity to test the effect of LTO on investment performance. Our approach takes advantage of emerging online media resulting in limited length of the observation period. The author was only able to collect LTO data in the past few years; hence, the time window to observe investment performance is insufficient, as it takes years to see whether the investment can be paid back (Drover et al., 2017). Nevertheless, whether LTO can bring VCs with better investment performance is a research topic worthy of investigation. Another rising force is CVC with dual motivations for industrial and financial goals. Investigation on CVCs will reveal resonant effects of the psychological mechanism on both the strategic and financial decisions compared with independent VCs focusing more on financial goals. Meanwhile, the approach of this study can also be applied to similar frameworks about start-ups to investigate the impacts of LTO or other psychological factors on performance at the organizational level. Lastly, studies on venture investment always treat the formation of investment ties as a unilateral decision, made by either investors or entrepreneurs. The proven similarity bias exiting in a venture investment suggests more extensive “matching” effects between ventures and investors. Hence, future researchers are encouraged to explore “matching” effects under psychological frameworks.

## Data Availability Statement

The original contributions presented in the study are included in the article/supplementary material, further inquiries can be directed to the corresponding author/s.

## Author Contributions

The author designed this study, conducted the empirical test and completed the writing.

## Conflict of Interest

The author declares that the research was conducted in the absence of any commercial or financial relationships that could be construed as a potential conflict of interest.

## Publisher’s Note

All claims expressed in this article are solely those of the authors and do not necessarily represent those of their affiliated organizations, or those of the publisher, the editors and the reviewers. Any product that may be evaluated in this article, or claim that may be made by its manufacturer, is not guaranteed or endorsed by the publisher.

## Footnotes

1 WeChat launched the official account function in 2012.

2 The simplified Chinese dictionary of LIWC is a translated version of the official English dictionary of LIWC and takes the nuances of the Chinese language into consideration. It is officially offered by LIWC.
